# Perception of cure among patients with metastatic genitourinary cancer initiating immunotherapy

**DOI:** 10.1186/s40425-019-0557-5

**Published:** 2019-03-12

**Authors:** Cristiane Decat Bergerot, Paulo Gustavo Bergerot, Errol J. Philip, Jo Ann Hsu, Nazli Dizman, Ulka Vaishampayan, Tanya Dorff, Sumanta Kumar Pal

**Affiliations:** 1 Department of Medical Oncology & Experimental Therapeutics, City of Hope Comprehensive Cancer Center, 1500 East Duarte Road, Duarte, CA 91010 USA; 2 University of California San Francisco, San Francisco, CA USA; 3 Department of Oncology, Wayne State University/Karmanos Cancer Institute, Detroit, MI USA

**Keywords:** Genitourinary cancers, Decision making, Perception of prognosis, Treatment outcomes understanding

## Abstract

**Background:**

Despite the advent of checkpoint inhibitors (CPIs) for advanced genitourinary (GU) cancers, existing studies suggest that durable complete responses are observed in fewer than 10% of patients. This study sought to evaluate the association between expectations of cure reported by patients with advanced GU cancers initiating immunotherapy and quality of life (QOL), anxiety and depression.

**Patient and methods:**

A single-institution, cross-sectional survey study was conducted with patients preparing to receive CPIs for treatment of metastatic renal cell carcinoma (RCC), urothelial cancer (UC) and prostate cancer (PC). Patients were assessed prior to initiation of immunotherapy for expectations of cure (divided into four quartiles), quality of life (QOL; Functional Assessment of Chronic Illness Therapy-General [FACT-G]), and symptoms of anxiety and depression (PROMIS).

**Results:**

Sixty patients were enrolled, with metastatic RCC, UC and PC comprising 63, 28 and 8% of the study population, respectively. Median age of the cohort was 65 (range, 31–91), and 68% were male; 33% received CPI in the first-line setting. Despite extensive counseling from oncologists regarding potential clinical outcomes with immunotherapy, a substantial proportion of patients (23%) harbored inaccurate expectations regarding the potential benefit of immunotherapy. Importantly, patients with accurate expectations of cure reported lower anxiety scores using the PROMIS-Anxiety inventory. No significant differences were found between expectations of cure and quality of life or depression, using the FACT-G and PROMIS-Depression inventories, respectively.

**Conclusion:**

The current study found that a considerable proportion of patients with advanced GU cancers harbor inaccurate expectations concerning the potential benefit of immunotherapy. These results suggest that more effective counselling may mitigate patient anxiety, and potentially promote treatment satisfaction and adherence.

## Introduction

The advent of immunotherapy has brought remarkable improvements in treatment strategies for metastatic genitourinary cancer. Data from CheckMate-214 demonstrated an impressive improvement in overall survival with nivolumab/ipilimumab versus sunitinib, with 9% of patients with metastatic renal cell carcinoma (mRCC) achieved a complete response with nivolumab/ipilimumab [1]. Several further trials have shown promising results for metastatic urothelial carcinoma (mUC). Complete responses were achieved in 7% of patients treated with pembrolizumab (KEYNOTE-052) and 9% with atezolizumab (IMvigor210) [2, 3]. More recently, studies have shown durable responses with pembrolizumab for patients with metastatic prostate cancer (mPC), albeit with a sobering objective response rate of 6% [4, 5]. With these advances in treatment, it is important that patients are counseled and understand that cure, or a durable complete response, is still relatively uncommon.

Despite the expansion of treatment options and more widespread use of immunotherapy, little is known regarding patient perceptions of this new modality. It is unclear whether patients possess an accurate understanding of the clinical benefits associated with these agents. We hypothesize that patients may overestimate the potential benefits of immunotherapy, partly due to the well-publicized complete responses that have been documented in the lay press. The current study aims: [1] to determine the proportion of patients who anticipate cure with immunotherapy for metastatic genitourinary cancer, and [2] to evaluate the association between expectations of cure and quality of life (QOL), anxiety and depression.

## Methods

A single institution survey assessed patient perceptions regarding prognosis with immunotherapy. Patients with metastatic genitourinary cancer were enrolled from October 2017 to October 2018, and were treated by any one of three genitourinary medical oncologists practicing at the institution. Patients were eligible if they had mRCC, mUC or mPC and were about to embark on checkpoint inhibitors. Data were collected prior to administration of immunotherapy, but only after patients received formal extensive counseling from their oncologists that addressed predetermined topics including standard of care, treatment options and goals of treatment. Patients who consented responded to a survey and sociodemographic and clinical data (e.g., age, gender, marital status, education level, annual income, diagnosis, and treatment) was extracted from medical records. This study was approved by the IRB.

### Measures

The survey administered was comprised of four parts. First, patients were asked if they felt: [1] cure is very likely and is in the range of 76 to 100%, [2] cure is likely and is in the range of 51 to 75%, [3] cure is possible but not likely and is in the range of 26 to 50%, or [4] cure is not at all likely and is in the range of 0 to 25%. For purposes of the survey, cure was equated to a durable complete response. QOL was assessed using the Functional Assessment of Chronic Illness Therapy-General (FACT-G) scale, comprised of 27-items (item range 0 to 4), scored from 0 to 108, assessing physical, social/family, emotional and functional well-being [6]. Anxiety was assessed using the PROMIS-Anxiety scale, consisting of 8 self-reported items scored from 1 to 5 points that assess symptoms of anxiety (e.g., fear, anxious misery, hyperarousal, and somatic symptoms related to arousal) over the past seven days [7]. Depression was assessed using the PROMIS-Depression scale, which assesses 8 self-reported items (range from 1 to 5 points [7]. The items included were negative mood, decrease in positive affect, information-processing deficits, negative views of the self, and negative social cognition.

### Analysis

Logistic regression was used to explore patient’s characteristics associated with expectation of cure. Linear regression adjusted for confounders were run to test the association between QOL, anxiety and depression (dependent variable), and expectation of cure. For both analyses, expectation of cure was defined as accurate (cure estimates from 0 to 25%) and inaccurate (cure estimates greater than 25%).

## Results

Sixty of the 64 patients approached were enrolled (Table 1). Patients were an average of 65 years old (SD = 13; range from 31 to 91), and the majority were male (68%), married (81%), Caucasian (75%), and highly educated (58% college degree). Most respondents were not currently working (48% retired, 26% disabled), and 55% possessed an annual income greater than $100,000 USD. Types of cancer included mRCC (63%), mUC (28%) and mPC (8%). Nivolumab (35%) and atezolizumab (30%) were the most frequent types of immunotherapy reported, and were administered as first (33%) or second line of therapy (45%) for the majority of patients. Notably, although genomic profiling was not consistently performed in the cohort, no patients in the present study received immunotherapy contingent upon the presence of microsatellite instability.

**Table 1:** Sociodemographic and clinical characteristics of patients with metastatic genitourinary cancer (*N* = 60)

Characteristics	*N* (%)
Gender	
Male	41 (68.3)
Female	19 (31.7)
Age	
M (SD; min-max)	65.1 (13.1; 31–91)
Marital Status	
Single	3 (5.0)
Married	49 (81.7)
Divorced	3 (5.0)
Widowed	5 (8.3)
Education	
Elementary School	4 (6.7)
High School	5 (8.3)
Some College	16 (26.7)
College Degree	23 (38.3)
Beyond College	12 (20.0)
Race	
White	45 (75.0)
Hispanic	5 (8.3)
Black	2 (3.3)
Japanese	3 (5.0)
Chinese	2 (3.3)
East Asian	2 (3.3)
South East Asian	1 (1.7)
Annual Income	
Less than 40,000	4 (6.7)
40,000 to 100,000	23 (38.3)
More than 100,000	33 (55.0)
Employment Status	
More than 32 h	6 (10.0)
Less than 32 h	6 (10.0)
Unemployed	1 (1.7)
Homemaker	2 (3.3)
Disability	16 (26.7)
Retired	29 (48.3)
Cancer	
Renal Cell Carcinoma	38 (63.4)
Urothelial Carcinoma	17 (28.3)
Prostate Cancer	5 (8.3)
Immunotherapy	
Nivolumab	21 (35.0)
Atezolizumab	18 (30.0)
Nivolumab/Ipilimumab	13 (21.7)
Pembrolizumab	6 (10.0)
Avelumab	2 (3.3)
Line of Therapy	
1st line	20 (33.3)
2nd line	27 (45.0)
3rd line	6 (10.0)
4th line	4 (6.7)
5th line	3 (5.0)

In general, 23% of patients endorsed the inaccurate belief that cure was “very likely”, with a likelihood of cure from 76 to 100%. Consistent with existing evidence, 71% of patients (71% diagnosed with mRCC, 70% with mUC and 80% with mPC) considered cure to be “not at all likely” (0 to 25%). A similar mean QOL score was reported by patients with accurate expectations of cure compared to those with inaccurate beliefs (M = 88 vs. M = 86; *P* = 0.86). Notably, a greater proportion of patients with accurate expectations of cure reported lower prevalence of anxiety compared to those with inaccurate expectations (48% vs 82%, *P* = 0.01). There were no significant differences in depression scores (29% vs 28% respectively, *P* = 0.57).

Using logistic regression, older age and higher annual income were significantly associated with accurate expectations of cure (Table 2). Further, a linear regression analysis revealed a significant association between lower anxiety scores and more accurate expectations of cure (Table 3).

**Table 2:** Summary of Logistic Regression for sociodemographic variables predicting expectations of cure

Sociodemographic characteristics	Multivariate association with accurate expectations of cure		
	OR (SE)	95% IC	*P*-value
Age	1.09 (0.03)	1.03 to 1.16	0.003
Gender	2.76 (0.71)	0.68 to 11.12	0.15
Marital Status	1.11 (0.70)	0.39 to 6.31	0.51
Education	0.68 (0.59)	0.21 to 2.20	0.53
Race	0.89 (0.67)	0.24 to 3.32	0.86
Annual Income	2.41 (0.46)	0.96 to 6.02	0.04

**Table 3:** Summary of Linear Regression Analysis for patient reported outcomes predicting expectations of cure

Patient Reported Outcomes	Multivariate association with expectations of cure		
	B (SE)	95% IC	*P*-value
Quality of Life	1.69 (3.99)	−6.29 to 9.69	0.67
PROMS Anxiety	−1.99 (0.77)	−3.76 to −1.12	0.01
PROMS Depression	−0.05 (2.28)	−4.62 to 4.51	0.98

## Discussion

To our knowledge, this is the first study to examine expectations of cure among patients with metastatic genitourinary cancer who received immunotherapy. A substantial proportion of patients (23%) harbored inaccurate expectations concerning the potential benefit of immunotherapy, even after counseling from their oncologist. This finding is consistent with previous studies that have shown that many patients possess a more optimistic, albeit less accurate, perception regarding their prognosis [8]. Inaccurate perceptions regarding treatment and its benefits can create barriers to informed decision making and promote dissatisfaction with treatment outcomes [9]. The association between accuracy in perceptions of prognosis and psychosocial well-being have been explored in other settings, including metastatic lung cancer and colorectal cancer [9–13], but have yielded variable findings, with some studies suggesting that an accurate perception of prognosis may enhance QOL while others report just the opposite.

Our preliminary findings suggest that an accurate expectation of cure was associated with older age and higher income. Prior research has demonstrated that younger and less educated patients may be less involved in medical decision making and thus possess a poorer understanding of their treatment, as well as being less satisfied with the care received [14]. A further notable finding was the association between accurate expectation of cure and lower rates of anxiety, suggesting that counseling that promoted accurate patient understanding of immunotherapy could be associated with improved psychosocial outcomes. Further research is needed to assess exactly what aspects of counseling can be effective in promoting accurate patient understanding. As a corollary to this, psychological therapies (e.g., cognitive behavioral therapy) can mitigate negative intrusive thoughts patterns (realistic concerns versus cognitive distortions) and thus enable patients to cope better with their current health status, make informed treatment decisions, and prepare for their future.

Several limitations should be noted. First, the sample size was small and not representative of the general population (patients were mostly well-educated and with high levels of income), thus preventing definitive conclusions regarding the association between expectation of cure and patient reported outcomes. Second, patients were recruited from a single institution and within this cohort, three separate tumor types were assessed with a multitude of different checkpoint inhibitors. Future studies should validate these findings and assess these parameters longitudinally in a multicenter study, thus providing insight into more homogenous cohorts with regard to cancer type**,** checkpoint inhibitor treatment, and volume of metastatic disease. Importantly, an extension of the current study is planned in an upcoming SWOG trial for patients with mRCC receiving first line immunotherapy.

These limitations notwithstanding, the current study provides unique insight into patients’ perceptions of immunotherapy. These results suggest that a population of patients exist who may need more extensive counseling regarding treatment options and the benefits of immunotherapy. With the emergence of multiple new immunotherapy modalities, such as adoptive cellular approaches and vaccines, it is likely patients will face even more challenges in understanding treatment options and prognosis, and thus the development of effective counseling approaches should be a priority.
